# Successful Treatment of Extragenital Lichen Sclerosus With Roflumilast 0.3% Cream: A Report of Two Cases

**DOI:** 10.7759/cureus.100823

**Published:** 2026-01-05

**Authors:** Serena Dienes, Muhammad Mahmood, Marlene Dytoc

**Affiliations:** 1 Temerty Faculty of Medicine, University of Toronto, Toronto, CAN; 2 Department of Medicine, Faculty of Medicine and Dentistry, University of Alberta, Edmonton, CAN; 3 Department of Laboratory Medicine and Pathology, University of Alberta, Edmonton, CAN; 4 Division of Dermatology, Department of Medicine, University of Alberta, Edmonton, CAN

**Keywords:** extragenital lichen sclerosus, extragenital lichen sclerosus et atrophicus, genital lichen sclerosus, lichen sclerosus, lichen sclerosus atrophicus, lichen sclerosus et atrophicus, roflumilast

## Abstract

Extragenital lichen sclerosus (EGLS) is often resistant to first-line ultrapotent corticosteroids, with few steroid-sparing options available. Topical roflumilast has been approved by the US Food and Drug Administration (FDA) for an expanding number of indications in dermatology, including plaque psoriasis, atopic dermatitis, and, most recently, seborrheic dermatitis. We present two cases of symptomatic EGLS, who both demonstrated clinical improvement using roflumilast 0.3% cream as early as one month of follow-up.

## Introduction

Lichen sclerosus (LS) is a chronic, inflammatory cutaneous condition most commonly seen in post-menopausal females that is usually confined to the anogenital region [1]. The pathogenesis of LS is not fully understood, though increased expression of Th1-specific pro-inflammatory cytokines and upregulation of BIC/microRNA-155 are seen in certain autoimmune phenotypes [2]. Genetic predisposition and altered hormonal composition could also play a role [3]. Extragenital lichen sclerosus (EGLS), comprising 15% of all LS cases, presents with white, “cigarette paper” textured papules and plaques, surrounding erythema, and progressive atrophy and sclerosis with a predilection for the upper body [1]. It often manifests in conjunction with anogenital involvement [1]. Histologically, EGLS evolves from an interface dermatitis in the early pre-sclerotic/inflammatory stage, to hyalinization and a deeper dermal lymphocytic infiltrate in the sclerotic stage, to epidermal atrophy, sclerosis, and minimal infiltrate in late disease [4,5]. Unlike anogenital LS, EGLS is frequently asymptomatic, is not typically associated with a malignant transformation to squamous cell carcinoma, and therefore, can often be monitored without treatment [6]. However, symptomatic EGLS can present with pruritus that ranges from mild to debilitating and is traditionally challenging to treat. We present two cases of EGLS that demonstrated physical and symptomatic improvement as early as one month after initiation of roflumilast 0.3% cream.

## Case presentation

Verbal consent was obtained from both patients. This study was exempt from institutional review board review by the Research Ethics Board (REB), as per institutional guidelines.

Patient 1

**Figure 1:**
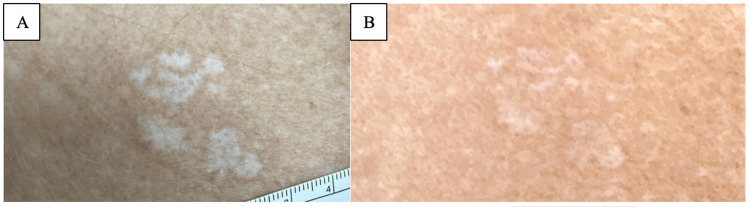
Extragenital lichen sclerosus on the right posterior shoulder of patient 1 before and after treatment with roflumilast 0.3% cream. Well-demarcated white atrophic papules and plaques with telangiectases on the right posterior shoulder of patient 1 before (A) and after (B) treatment with roflumilast 0.3% cream.

**Figure 2:**
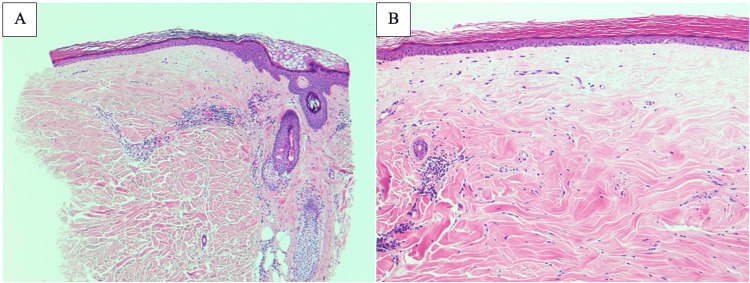
Histopathologic confirmation of extragenital lichen sclerosus on the mid-back of patient 1. A: Sections of biopsy from mid-back displayed hyalinization of the upper dermis with underlying mild lymphocytic infiltrate (H&E stain, x50). B: Epidermis was attenuated with superficial dermal hyalinization (H&E stain, x100).

A 72-year-old Caucasian female with a 40-year history of biopsy-proven vulvar LS, a 10-month history of clinical EGLS, and osteoporosis presented for follow-up at our vulvar dermatology clinic in July 2025. Her current medications included clobetasol 0.05% ointment, intravaginal estrogen cream twice weekly for genitourinary syndrome of menopause, and vitamin D supplementation. Her vulvar LS was well-controlled on maintenance clobetasol 0.05% ointment three times weekly. However, while instructed to use clobetasol twice weekly for her EGLS, she reported as-needed application to her extra-genital lesions since their onset, and they remained intensely pruritic. Upon physical examination, she had well-demarcated hypopigmented plaques with telangiectases extensively spanning the right posterior shoulder, left midback, midline lower back, and left posterior neck (Figure 1). A biopsy of the left mid-back was performed, which showed a compact stratum corneum, epidermal atrophy, flattening of rete ridges, superficial dermal papillary homogenization, and an underlying mild lymphocytic infiltrate (Figure 2). These findings, plus the coexistence of LS, aided the diagnosis of EGLS. While LS and morphea overlap disorder has been described, features of morphea (square punch, dense collagen bundles, “trapped” eccrine glands, deeper lymphocytic infiltrate) were not seen, and was thus ruled out.

The patient was interested in other treatment options for EGLS. She had previously failed tacrolimus 0.1% ointment and also had concerns about striae and systemic absorption with prolonged clobetasol 0.05% ointment use. Given that the patient did not live in proximity to a phototherapy suite and her osteoporosis made systemic steroids a less attractive option, we offered her the choice of roflumilast 0.3% cream daily or weekly methotrexate for her EGLS. She opted for a trial of topical roflumilast 0.3% cream daily, while continuing two to three times weekly application of clobetasol 0.05% ointment to the vulva. At one-month follow-up, the pruritus associated with her EGLS was improved with roflumilast, and there was a reduction in hypopigmentation and lesional diameter on exam. She did not experience any adverse reactions.

Patient 2

**Figure 3:**
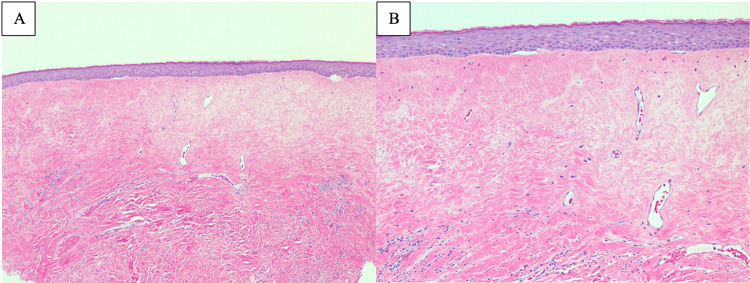
Histopathologic confirmation of lichen sclerosus on the vulva of patient 2. A: Sections of vulvar biopsy showed hyalinization of the upper dermis with sparse lymphocytic infiltrate (H&E stain, x50). B: Epidermis was attenuated with dermal hyalinization and telangiectatic vascularity (H&E stain, x100).

**Figure 4:**
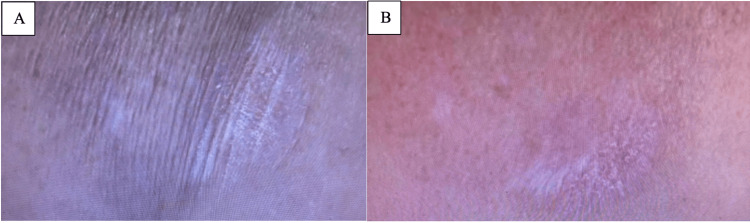
Extragenital lichen sclerosus on the right shoulder of patient 2 before and after treatment with roflumilast 0.3% cream. Well-defined pearly annular plaques with wrinkled skin on the right shoulder of patient 2 before (A) and after (B) treatment with roflumilast 0.3% cream.

In July 2025, a 76-year-old female presented to our vulvar dermatology clinic for follow-up of biopsy-proven anogenital LS (Figure 3) and clinically diagnosed EGLS. Her past medical history was otherwise significant for vitiligo, hypothyroidism, and depression. At that time, she was applying clobetasol 0.05% ointment three times weekly to the vulva, perianal area, and extragenital areas of involvement, which included her right upper arm, central chest, and left hip. Like our first patient, her EGLS was comparatively more active than her anogenital LS, and she also had concerns regarding long-term ultrapotent steroid use. Previous treatments included intralesional triamcinolone 5 mg/mL into particularly indurated extragenital annular plaques, as well as an erosion in the posterior fourchette the year prior. On physical examination, she had well-defined pearly annular plaques with wrinkled skin on the right upper arm and shoulder, abdomen, and hips. The vulva and perianal region had atrophic white plaques noticed in a figure-of-eight distribution, and there was significant resorption of the labia minora. We prescribed a tapering course of mometasone 0.1% ointment to be applied twice daily to the vulva, and roflumilast 0.3% cream to be applied daily to the perianal and extragenital areas. At one-month follow-up in August 2025, the patient revealed that she had been using roflumilast 0.3% cream twice daily, and her perianal and extragenital sites felt significantly less inflamed. This correlated with her physical exam findings, which demonstrated a reduction in lesion diameter and improvement in the appearance of wrinkling and atrophy of extragenital sites (Figure 4). She is continuing twice-daily application and has not experienced any side effects.

A comparison of clinical characteristics and outcomes between these two patients is provided in Table 1.

**Table 1:** Comparison of patient characteristics and outcomes. EGLS, extragenital lichen sclerosus; F, female.

Parameter	Patient 1	Patient 2
Age/sex	72/F	76/F
Comorbidities	Osteoporosis	Vitiligo, hypothyroidism, depression
EGLS duration before roflumilast 0.3% cream	10 months	13 years
Baseline clinical findings	Pruritic, well-demarcated hypopigmented plaques with telangiectases extensively spanning the right posterior shoulder, left midback, midline lower back, and left posterior neck	Pruritic, well-defined pearly annular plaques with wrinkled texture on the right upper arm, central chest, and left hip
Roflumilast 0.3% cream regimen	Once daily	Twice daily
Histopathologic findings of EGLS	Compact stratum corneum, epidermal atrophy, flattened rete ridges, superficial dermal papillary homogenization, and mild lymphocytic infiltrate	Not performed
Adverse events	None	None
Clinical findings at 1-month follow-up	Reduction in pruritus, hypopigmentation, and lesional diameter	Reduction in pruritus, lesion diameter, and improvement in the appearance of wrinkling and atrophy

## Discussion

Compared to anogenital LS, EGLS tends to be less responsive to ultrapotent topical steroids such as clobetasol 0.05% ointment, which is considered first-line treatment for all forms of LS. Furthermore, while the risk of skin atrophy and hypothalamic-pituitary-adrenal axis suppression is rare unless long-term application over a large body surface area is used, “steroid phobia” can significantly impair patients’ interest and adherence to treatment [7]. Topical calcineurin inhibitors can also be used, but can cause application site discomfort and have lower efficacy compared to topical steroids [8]. Systemic options for widespread disease include ultraviolet A1 (UVA1) phototherapy, methotrexate, or systemic steroids, though evidence is limited [9-11]. There is a need for more topical steroid-sparing agents that can be used in the management of EGLS, with greater efficacy during acute flares, improved safety as maintenance therapy, or for those with contraindications to systemic therapy.

Roflumilast is a selective, small-molecule inhibitor of phosphodiesterase-4 (PDE-4) whose mechanism of action involves competitive inhibition of PDE-4 cyclic adenosine monophosphate (cAMP) hydrolase. In preventing the breakdown of cAMP, roflumilast downregulates the production of proinflammatory cytokines in Th1 and Th17 cells [12]. It has been approved by the US Food and Drug Administration (FDA) for use in plaque psoriasis (including intertriginous areas) and atopic dermatitis as a cream, and its foam formulation is approved for those with scalp and body plaque psoriasis or seborrheic dermatitis. Across clinical trials, topical roflumilast demonstrated a favorable safety profile with excellent tolerability [13-15].

The rationale for using a PDE-4 inhibitor in our patients is supported by a growing body of evidence demonstrating the efficacy of this drug class in LS, as well as other lichenoid and inflammatory dermatoses. In a 2023 study, crisaborole 2% ointment, a less potent topical PDE-4 inhibitor, was reported in the successful treatment of "vulvar leukoplakia," a clinical term encompassing vulvar LS [16]. In this study, crisaborole 2% ointment demonstrated statistically significant improvement in lesion scores and symptoms compared to control after just two weeks, providing the first direct evidence for the utility of a topical PDE-4 inhibitor in a related clinical context [16]. Fage and Johansen reported a case of recalcitrant erosive genital lichen planus in which oral roflumilast allowed for the successful reduction of prednisone with three months of use [17]. Apremilast, an older oral PDE-4 inhibitor, has also been successfully used to treat recalcitrant cases of oral and cutaneous lichen planus, and randomized controlled trials evaluating apremilast for genital erosive lichen planus are ongoing [18]. However, like crisaborole, apremilast is also less potent than roflumilast, with an IC50 of 140 nM for apremilast versus 0.7 nM for roflumilast in vitro studies [19].

More recently, Domingues reported the case of a female with EGLS whose erythema reduced and pruritus fully resolved within three months of daily roflumilast 0.3% cream [20]. Like our first patient, this patient was also 72 years of age with reservations about long-term ultrapotent topical steroid use. However, unlike both of our patients, her EGLS was limited to a single atrophic plaque on the thigh, was treatment naïve, and did not have concomitant vulvar involvement. The patients presented in this report expand on Domingues’ findings of the efficacy of roflumilast 0.3% cream in EGLS, and that improvement may be seen as early as one month of use. While both of our patients demonstrated improvement, the discrepancy in roflumilast 0.3% cream application frequency warrants consideration. In the DERMIS-I, DERMIS-II, INTEGUMENT-I, INTEGUMENT-II, and ARRECTOR clinical trials, roflumilast was investigated with once-daily dosing [13-15]. In the 2023 study investigating crisaborole 2% ointment for vulvar leukoplakia, two patients (of 50) experienced local application site pain and ulceration. To date, no studies have investigated the safety or efficacy of twice-daily application of roflumilast 0.3% cream for LS or EGLS; however, our patient did not experience any application site discomfort.

## Conclusions

This case report is limited by short follow-up durations. Nevertheless, these cases illustrate the potential role of roflumilast 0.3% cream as a well-tolerated, steroid-sparing option for EGLS, with efficacy in those who have failed potent and/or ultrapotent topical steroids. Larger, placebo-controlled studies with pre- and post-application site biopsies would be beneficial in supporting these clinical observations and in investigating the potential role of twice daily roflumilast application.
